# Low intensity vs. self-guided Internet-delivered psychotherapy for major depression: a multicenter, controlled, randomized study

**DOI:** 10.1186/1471-244X-13-21

**Published:** 2013-01-11

**Authors:** Yolanda López-del-Hoyo, Barbara Olivan, Juan V Luciano, Fermín Mayoral, Miquel Roca, Margalida Gili, Eva Andres, Antoni Serrano-Blanco, Francisco Collazo, Ricardo Araya, Rosa Baños, Cristina Botella, Rosa Magallón, Javier García-Campayo

**Affiliations:** 1 Departamento de Psicología y Sociología, Universidad de Zaragoza, Zaragoza, Spain; 2 Parc Sanitari Sant Joan de Déu and Fundación Sant Joan de Déu, Sant Boi de Llobregat, Barcelona, Spain; 3 Psychiatric Service, University Hospital Carlos Haya, Malaga, Spain; 4 Institut Universitari d'Investigació en Ciències de la Salut (IUNICS), University of Balearic Islands, Palma de Mallorca, Spain; 5 Unidad Epidemiología Clínica, Hospital 12 de Octubre, CIBER Epidemiología y Salud Pública, Madrid, Spain; 6 Servei de Psiquiatria, Hospital Universitari Vall d'Hebron, Barcelona, Spain; 7 Academic Unit of Psychiatry, School of Social and Community Medicine, University of Bristol, Bristol, UK; 8 Universidad de Valencia, Madrid, Spain; 9 Universitat Jaume I, Castellon, Spain; 10 Ciber Fisiopatología de la Obesidad y Nutrición (CB06/03), Instituto de Salud Carlos III, Madrid, Spain

**Keywords:** Depression, Computer-delivered psychotherapy, Randomized controlled trial

## Abstract

**Background:**

Major depression will become the second most important cause of disability in 2020. Computerized cognitive-behaviour therapy could be an efficacious and cost-effective option for its treatment. No studies on cost-effectiveness of low intensity vs self-guided psychotherapy has been carried out. The aim of this study is to assess the efficacy of low intensity vs self-guided psychotherapy for major depression in the Spanish health system.

**Methods:**

The study is made up of 3 phases: 1.- Development of a computerized cognitive-behaviour therapy for depression tailored to Spanish health system. 2.- Multicenter controlled, randomized study: A sample (N=450 patients) with mild/moderate depression recruited in primary care. They should have internet availability at home, not receive any previous psychological treatment, and not suffer from any other severe somatic or psychological disorder. They will be allocated to one of 3 treatments: a) Low intensity Internet-delivered psychotherapy + improved treatment as usual (ITAU) by GP, b) Self-guided Internet-delivered psychotherapy + ITAU or c) ITAU. Patients will be diagnosed with MINI psychiatric interview. Main outcome variable will be Beck Depression Inventory. It will be also administered EuroQol 5D (quality of life) and Client Service Receipt Inventory (consume of health and social services). Patients will be assessed at baseline, 3 and 12 months. An intention to treat and a per protocol analysis will be performed.

**Discussion:**

The comparisons between low intensity and self-guided are infrequent, and also a comparative economic evaluation between them and compared with usual treatment in primary. The strength of the study is that it is a multicenter, randomized, controlled trial of low intensity and self-guided Internet-delivered psychotherapy for depression in primary care, being the treatment completely integrated in primary care setting.

**Trial registration:**

Clinical Trials NCT01611818

## Background

Major depression is a prevalent mental disorder associated with significant disability and economic costs [1]. In this sense, it is known that 25% of all human beings will suffer from a depression at any moment over their lives [2] and, according to the World Health Organization [3], depression will become the second most important cause of disability in 2020. From the perspective of the health system, 25-35% of the patients attending primary care settings suffer from a psychiatric disorder and more than 80% of them are depression and/or anxiety [4]. This percentage is higher at the Spanish Primary Care where 45% of the patients have had a mental disorder during their life, with 31% experiencing a mental disorder in the past 12 months [5]. Almost 30% attendees reported a lifetime history of major depressive disorder, with 9.6% experiencing major depression in the past year. In most countries, general practitioners refer only 5-10% of the patients with psychiatric disorders to specialized services [4] and, despite of that, mental health services are collapsed all over the world.

Major depression can be treated effectively using antidepressants, but relapse is high following cessation, and many patients prefer psychological therapies [6], which are as effective as pharmacological treatment [7]. Although evidence-based treatments exist, rates of treatment seeking are low, and many patients with depression do not receive adequate management, being the main barriers the limited availability of trained clinicians with consequent long waiting lists, direct and indirect costs of treatment, stigma, and difficulty attending therapy during business hours [8]. These reasons, expected to get worse in the future, had convinced international health authorities to search for new cost-effective treatment alternatives for depression [9].

It has been defined “Computer-delivered psychotherapy” (CDP) as any psychotherapy program that uses patients’ inputs to take decisions regarding treatment [10-12]. This excludes videoconferences, self-help programs exclusively based on bibliotherapy, chats, help groups, etc. Patients receive therapy using their computers at home and the sessions are usually short (20–30 min.), on a weekly basis and the treatment lasts 3–6 months [10-12]. At this moment, there exist evidence of the effectiveness of CDP in depression [13], anxiety [14], and other psychiatric disorders such as alcohol abuse, psychosomatic disorders [15] or even pain [16]. Recently, cost-effectiveness studies on CDP have been published with satisfactory results [17-20]. The results has lead to British NICE to support the use of a computer-delivered psychotherapy program for the treatment of depression ("Beating the Blues") to be widely used by the patients of the British National Health Service [11,21]. Other studies show that CDP is so effective that it should be used not only at primary care level, but even in mental health services. In these specialized settings, it would be recommended as a self-help first step for depression and anxiety, before being attended by a psychologist or psychiatrist [22]. Recently, other CDP programs without the presence of a psychotherapist, such as "Blues Begone", have been evaluated with adequate effectiveness not only in randomized controlled trials [23] but in naturalistic studies as well [24].

Internet-delivered psychotherapy (iPT) is a form of CDP delivered by internet. According to NICE [21], iPT programs are highly structured and comprises systematically presented online lessons, homework, and supplementary resources. Programs may be entirely self-guided, or patients may receive therapist contact via asynchronous e-mails or synchronous online chat or telephone calls. Depending on the time devoted by the psychotherapist to the patient, iPT programs can be broadly divided into low-intensity (<3 h) and high-intensity iPT (>3 h of therapist time in total) [25]. Although an Internet connection and basic computer hardware are required, some of the advantages of iPT include convenience, treatment fidelity, and accessibility. That is, patients and therapists may logon to the program at anytime, every patient receives exactly the same materials, and barriers relating to stigma, geography and limited therapist resources are minimized [21].

While there is consensus about the effectiveness of iPT for major depression, it is still unknown how these interventions work and for whom they work. This holds true for other types of intervention as well, despite some exploratory studies on this subject [26,27]. For this reason, it is relevant to examine potential mediating and moderating variables that explain the effect of these treatments. Another key challenge is how to successfully adapt and disseminate iPT programs developed and evaluated in a research environment to the heterogeneous health services of developed and developing countries. One recent example is a program that was effective in reducing depressive symptoms in an RCT [28], but which did not improve treatment outcomes when added to treatment as usual in primary care settings [29]. Regarding this, it is recommendable to develop studies in which iPT be integrated within usual primary care services. The efficacy and reliability of iPT have been demonstrated for depression in different countries and different languages, especially English, but as far as we know, there is no validated internet delivery program in Spanish. Since Spanish is, after English one of the most used languages in the world, it seems highly relevant that an online treatment program in Spanish for depressive patients in primary care could be tested. Finally, an unresearched question is the attitude towards iPT not only of the patients that receive it, but of the health professionals (both general practitioners and psychologists/psychiatrists) and stakeholders. Curiously, the attitudes of professionals are usually more negative than patients’ [30,31], but both are moderated by their experience with iPT [32,33]. To better tailor the programs to the environment in which they are intended to be used and about the acceptability to stakeholders, in order to facilitate the integration of these programs into the health system, qualitative studies to identify attitudes and barriers are systematically recommended.

The main objective of this study will be to compare the effectiveness of a low intensity vs. a self-guided Internet-delivered psychotherapy program compared with ITAU for the treatment of major depression in primary care in Spain, with a multicenter, randomized, controlled trial. The secondary objective will be the cost-effectiveness of those programs, to identify the patients that most benefit of these programs and to examine the potential mediators and moderators. In this article we describe the design of the study.

## Methods

### Study design

This study is a multicenter, randomized controlled trial. Subjects will be randomized into three groups: a) Low intensity Internet-delivered psychotherapy, b) Self-guided Internet-delivered psychotherapy and c) improved Treatment as Usual (ITAU) group in primary care. The evaluation of the treatment outcomes will be performed at patient level and they will be assessed individually.

### Setting and study sample

Patients will be recruited from primary health care centers of the three Spanish regions participating in the study. Patients will be recruited by general practitioners (GPs) working in these primary care centers until the required sample is completed, without a quota of patients assigned for each centre. Patients considered for inclusion are those aged 18–65 years, able to understand and read Spanish, with moderate or mild major depression, duration of symptoms longer than 2 weeks, access to Internet at home and having an email address. Exclusion criteria includes any psychological treatment during last year, severe psychiatric disorder in Axis I (alcohol/substances abuse or dependence, psychotic disorders or dementia) and patients with severe depression (indicated by a Beck-II score of 29 or higher) who will be advised to consult their GP. Receiving pharmacological treatment with antidepressants is not an exclusion criteria meanwhile, during the study period, treatment will not be modified or increased (decrease of pharmacological treatment is accepted). Diagnosis of major depression will be carried out with MINI International Neuropsychiatric Interview + scoring of moderate or mild depression using Beck Depression Inventory II. Cut-off point for this questionnaire is: 0–13: minimal depression; 14–19: mild depression; 20–28: moderate depression; 29–63: severe depression [34,35].

### Sample size

The sample size of this study was based on the expected difference on the primary outcome variable, i.e. depressive symptoms, between the intervention groups and the Treatment as Usual group at post-test. Based on a power of 0.80 in a one-tailed test, an alpha of 0.05, we need 100 subjects in each condition to show an effect-size of 0.40.

Therefore, the total sample size was determined at 300. This sample size also allows calculating clinically significant difference in the main outcome variable, Beck Depression Inventory II. Based on previous studies [36], this difference has been placed at 5 points. It allows changing scoring from normality to mild depression or from mild to moderate depression. Taking account an expected withdrawal rate of 30% according to previous studies [9-14], a sample size of 450 patients (150 by arm) was calculated. The three participating health centers will select 150 patients with cluster randomization to allocate one third in each of the three arms.

### Recruitment

Participants will be recruited in primary care settings by participating GPs among patients fulfilling study criteria. When the GP identifies a potential participant, he will explain the patient the characteristics of the study. Whether the patient is interested in participating he/she will sign an informed consent and the GP will fill a document describing the sociodemographic and clinical characteristics of the patient and will give him/her the patients’ information sheet and a handout describing the study. After giving informed consent, subjects will be interviewed in the next 3 days by a researcher. After confirming that have signed the informed consent and understand the study and the treatment options, the researcher will administer psychological tests related with inclusion criteria: MINI International Neuropsychiatric Interview and Beck Depression Inventory II. Patients in the ITAU arm will be allowed to follow psychotherapy program at the end of the study for ethical reasons. Whether the patient fulfills all study criteria, the researcher will administer the remaining baseline tests and will contact an independent researcher to implement randomization.

Recruitment will be done in a consecutive way up to complete sample size and it is expected to take a period of 12 months. Recruitment calculations have been carried out, on conservative bases, according to PREDICT data study [36] that describes a cumulated incidence of depression of 11.5% of the consulting population in primary care. That is to say that for every 1000 non-depressed consulting patients in primary care, 115 will get depressed over 1 year. Since the prevalence of depression is 14%, a GP with a standard consultation made up of 1500 patients, will have 210 patients diagnosed of major depression. From the remaining 1290 non-depressed patients, 148 will get depressed over 1 year, which stands for 2.8 patients a week or 12.3 patients a month. Assuming that every GP will invite 25% of those 12 possible patients and that only 25% will fulfill criteria to participate in the study and will give consent to it, every GP will recruit 1 patient/month during the estimated 12-month recruited period. This allows an estimation of at least 10 GPs for every region (Aragon, Balearic Islands and Andalucía).

### Randomization, allocation and masking of study groups

Each patient will be allocated to either one of the three groups using a computer-generated random number sequence. We will use block allocation, with 3 blocks (one for each participating region) including 150 patients per block, with about 50 patients per arm. The allocation will be carried out by an independent person, belonging to REDIAPP (Primary Care Prevention and Health Promotion Research Network), who is not involved in the study. The method used to implement the random allocation sequence will be a central telephone. The sequence will be concealed until interventions are assigned. Patients will agree to participate before the random allocation and without knowing which treatment they will be allocated to. Study personnel conducting psychological assessments will be masked to participants' treatment conditions.

**Figure 1:**
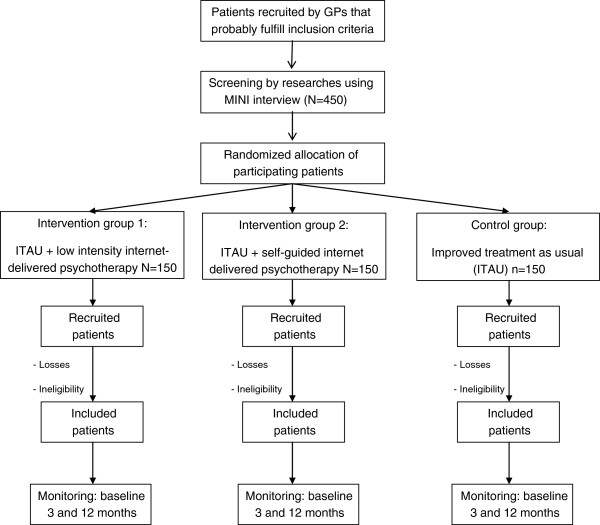
Flowchart of the study: randomization, sampling and monitoring of patients.

The researcher that administers baseline assessments will be unaware of the treatment group to which the patient belongs to. This researcher will be different from the one that administer the questionnaires over the study. GPs will be also unaware, as long as possible, of the arm to which the patient has been randomized, since their treatment should be exclusively based on the recommendations of the guides for the treatment of depression. The flowchart of the study is summarized in Figure 1 (Insert by here).

### Interventions

#### Internet-delivered psychotherapy

The program “Smiling is fun” is an internet-delivered, multimedia, interactive, self-help program for the treatment of depression, developed by our group based on similar programs in other countries that have demonstrated to be effective [37,38]. The treatment protocol is composed of 8 therapeutic modules and two initial modules (home and welcome). The therapeutic modules are oriented to work on different psychological techniques which will allow the individual to learn and practice adaptive ways to cope with depression and daily problems. Each module includes exercises to practice such techniques. These modules are sequential, in order to move step by step, all along the program. Duration of the program can vary among users, it is estimated that duration for most people will be 3 months. The content of the program is the following:

***M1. Motivation for change*** which aim is to analyze the advantages and disadvantages of changing, emphasizing the importance of being motivated, promoting the individual’s implication to practice and learn all the abilities the program introduces.

***M2. Understanding emotional problems:*** This module provides information to recognize and understand the emotional problems.

***M3. Learning to move on:*** This module focuses on behaviour activation by teaching the importance of “moving on” to acquire a proper level of activity and involvement in life.

***M4. Learning to be flexible:*** This module aims to teach a more flexible way of thinking and to interpret situations, in order to be able to seeing every situation from different perspectives, learning to think about different alternatives.

***M5. Learning to enjoy:*** This module is devoted to teach the importance and performance of positive experiences that generate positive emotions, promoting the involvement in pleasant and significant activities, and contact with other people.

***M6. Learning to live:*** This module involves a further step in the enhancement of positive affect, understanding the importance of identifying the individual’s own psychological strengths and selecting and carrying out meaningful activities linked to values and goals in life.

***M7. Living and learning:*** This module focuses on developing an action plan to boost the individual psychological strengths.

***M8. From now on, what else…?:*** This final module aims to go on and strengthen what has been learnt during the program.

The program recommends working on one module at least for one complete week. The program sends a reinforcing message when the user finishes each module and when the user performs the homework. On the other hand, the program sends an email to the user and to the clinician when more than two weeks between sessions have passed without using it. This message encourages the patient to continue working to benefit of the program.

Patients randomized to this treatment will be given a password to accede to the program at home. An adherence protocol using data from the program will be implemented to know which sessions and homework the patient has done, how much time has taken for every session and psychotherapy technique and the break he has taken in every session. The patients allocated to this intervention will be broken down in two different groups:

*Low intensity Internet-delivered psychotherapy.* Over the treatment period, a researcher trained in psychotherapy will contact with the patients to offer help (asking for any difficulties or problems to fulfil the program), and suggesting to end the therapy within the estimated period. The patient could ask questions or advice to the psychotherapists with a total maximum of <1 hour over the treatment period. They could also ask a technician any problem with the running program in order to solve technological problems.

*Self-guided Internet-delivered psychotherapy.* In this group there will not be any contact with any therapist over the treatment period. Only technical questions about the working of the program could be asked to a technician by mail.

Both groups of patients will receive treatment as usual improved by their GPs, as described in the next paragraph.

#### Improved Treatment as Usual at primary care level (ITAU)

All the patients included in the study (whether they receive psychotherapy or not) will be also treated by their GPs. In practice, ITAU in primary care is any kind of treatment administered by the GP to the patient with depression. However, it can be considered that the Treatment as Usual in primary care will be improved because the participating GP will receive a training program of 3 hours on a widely used Spanish Guide for the Treatment of Depression in Primary Care, which is based on the NICE recommendations for this subject [39]. This Guide describes the use of antidepressants in adequate doses and time of administration. In case of suicide risk, severe social dysfunction or worsening of symptoms, it is recommended to refer the patient to mental health facilities [40].

For the three groups the use of health and social services (health professionals, medications, social workers and other services) will be registered using the CSRI [41].

### Instruments

Patients will be assessed at baseline, posttreatment, and 3 and 12 months after inclusion. The study variables assessed are summarized in Table 1.

**Table 1:** Study variables

**Instrument**	**Assessment area**	**Time of assessment**	**Applied by**
MINI Neuropsychiatric interview [31,32]	Psychiatric diagnosis	Baseline	Researcher A
Beck II Depression Inventory [40,41]	Severity of depression	Baseline and follow-up sessions*	Researcher A (baseline)
			Researcher B (follow-up sessions)
Sociodemographic data	Gender, age, marital status, education, occupation, economical level	Baseline	Researcher A
SF-12 Health Survey [40,41]		Baseline and follow-up sessions*	Researcher A (baseline)
			Researcher B (follow-up sessions)
EQ-5D [42]	Health related quality of life	Baseline and follow-up sessions*	Researcher A (baseline)
			Researcher B (follow-up sessions)
CRSI [43]	Health and social services use	Baseline and follow-up sessions*	Researcher A (baseline)
			Researcher B (follow-up sessions)

Follow-up sessions: Postreatment, 3 and 12 months postreatment.

#### Main outcome

Severity of depressive symptomatology measured by Beck Depression Inventory II [34] using its Spanish validated version [35]. This is one of the most widely instrument used to evaluate severity of depression in pharmacological and psychotherapy trials. This questionnaire has been used because it is recommended to assess depression in primary care patients in which comorbidity with medical disorders is frequent.

#### Secondary outcomes

Socio-demographic variables. The following socio-demographic data will be collected: gender, age, marital status (single, married/relationship, separated/divorced, and widowed), education (years of education), occupation, economical level (in relation with Spanish minimum monthly salary that at the moment of the study was 640€).

Mini-International Neuropsychiatric Interview (MINI). This is a short structured diagnostic psychiatric interview that yields key DSM-IV and ICD-10 diagnoses [42]. MINI can be administered in a short period of time and clinical interviewers need only a brief training. The MINI has been translated and validated in Spanish [43].

EuroQoL-5D questionnaire (EQ-5D – Spanish version) [44]. Generic instrument of health-related quality of life. It has two parts: Part 1 records self-reported problems in each of five domains: mobility, self-care, usual activities, pain/discomfort and anxiety/depression. Each domain is divided into three levels of severity corresponding to no problems, some problems, and extreme problems, which allows obtaining a population-based preference score or societal index (SI). A total of 243 theoretically possible health states can be obtained and the SI is calculated on the basis of these health states. Values range from 1 (best health state) to 0 (death). However, this index may also provide negative values that correspond to health states perceived as worse than death. Utility scores for these health states were assigned using the readily available Spanish population tariffs [45]. Part 2 records the subject's self-assessed health on a VAS, a 10 cm vertical line on which the best and worst imaginable health states score 100 and 0, respectively.

Client Service Receipt Inventory – adapted (CSRI – Spanish version) [41,46]. Questionnaire for collecting information about use of healthcare and social care services and other economic impacts (such as time off work due to illness). The variant used in this study was designed to collect retrospective data on service utilization during the previous months after the last assessment. Data on baseline assess the previous three months before inclusion.

Overall Depression Severity and Impairment Scale [47]**:** OASIS consists of 5 items that measure the frequency and severity of anxiety, as well as level of avoidance, work/ school/home interference, and social interference associated with anxiety. The instructions orient the respondent to considerate wide range of anxiety symptoms (e.g., panic attacks, worries, flashbacks) when answering the questions, and the time frame is “over the past week”. Respondents select among five different response options for each item, which are coded 0–4 and summed to obtain a total score. A psychometric analysis of the OASIS in an undergraduate sample suggested that the scale was unidimensional and has adequate psychometric properties [47].

Overall Depression Severity and Impairment Scale [47]: ODSIS is a self-report measure consists of 5 items. In this case, evaluating experiences related to depression. ODSIS measure the frequency and severity of depression, as well as level of avoidance, work/ school/home interference, and social interference associated with depression. Respondents select among five different response options for each item, which are coded 0–4 and summed to obtain a total score. This instrument can also be used to assess severity and impairment associated with low mood. ODSIS assesses the frequency with which the person has been depressed, the intensity of symptoms, behavioral avoidance and functional impairment associated with depression. So far, there are not published studies examining the psychometric properties of this scale. Norman et al. [47] recommended use and interpret it the same way to the OASIS.

Positive and Negative Affect Scale (PANAS). PANAS [48] consists of 20 items that evaluate two independent dimensions: *positive affect* (PA) and *negative affect* (NA). The range for each scale (10 items on each) is 10 to 50. The instrument’s psychometric properties are quite satisfactory. It has a validated Spanish version [49].

Credibility/expectancy questionnaire [50]. This instrument is a quick and easy-to-administer scale for assessing treatment expectancy and rationale credibility. Credibility has been defined as how believable, convincing, and logical the treatment is, whereas expectancy refers to improvements that clients believe will be achieved [51]. This questionnaire derives the two predicted factors (cognitively based credibility and relatively more affectively based expectancy) that are stable across different populations [52]. The aspects that address these two scales relate to: 1) treatment rationale, 2) treatment satisfaction, 3) degree to which I would recommend to a friend who had the same problem, 4) extent to which is considered to be useful in the same case, 6) extent to which the intervention would be considered aversive. This scale has demonstrated adequate psychometric properties [52]. Our research team has used these scales repeatedly in previous studies with good results [53].

### Ethical aspects

Informed consent will be obtained from the participants before they are aware of which group they are to be included in. Before they give their consent, the patients will be provided with a general overview of the aims and characteristics of the study and the psychological and pharmacological intervention. They will also be informed that they will be participating voluntarily, and that they can choose to withdraw at any time with the guarantee that they will continue to receive the treatment considered most appropriate by their GP. The study follows Helsinki Convention norms and posterior modifications and the Declaration of Madrid of the World Psychiatric Association. For ethical reasons, patients allocated to Treatment as Usual will be offered the possibility to complete the psychotherapy program. The Study Protocol was approved by the Ethical Review Board of the regional health authority (ref: PI10/039).

### Analysis strategy

#### Analysis of clinical efficacy

The analysis will be per intent to treat and will follow CONSORT recommendations [54]. First, the three groups will be compared in order to verify that there are no significant differences among them at baseline to confirm they are comparable after randomization.

For comparisons, the ANOVA for continuous variables and the Chi-squared test for categorical variables will be used. Non-parametric tests may also be used. To confirm main hypothesis, an analysis of variance (ANOVA) of repeated measures, including all the assessments over the time, will be carried. The main outcome, scoring of BDI-II, will be used as continuous variable. Models will include adjustment by baseline value of BDI-II and any other variable that show differences among groups at baseline. Possible interactions Group × Time will be assessed using Mixed Factorial Anova (3 × 4: 3 groups by 4 temporal moments). Other linear regression models will be used to compare differences in BDI-II scoring among groups for each assessment over the time compared to baseline. Similar analysis will be carried out for secondary outcomes. For missing values a sensitivity analysis will be used to estimate the effect of missing values. Those valued will be replaced using different systems such as last registered values and imputations.

#### Descriptions of costing procedure

Costs will be estimated from the healthcare and societal perspective during the one year of follow-up. *Direct health care costs* will be calculated by adding the costs derived from medication consumption (antidepressants and anxyolitics), medical tests, use of health-related services, and cost of staff to run the intervention. The cost of medication will be calculated by determining the price per milligram during the study, according to the Vademecum International (Red Book; edition 2013), including value-added tax. Total costs of medications will be calculated by multiplying the price per milligram by the daily dose in milligrams and the number of days receiving such treatment. The main source of unit cost data related to public medical tests and use of health services will be provided by the tariffs published in the Official Government Board of each Autonomous Community participating in the study. Tariffs for private sector will be obtained in each region consulting the last published tariff and inflated to 2013 using national Consumer Price Index. *Indirect costs* will be calculated considering the days on sick leave and multiplying them by the minimum daily wage in Spain for 2013. Finally, total costs will be calculated by adding direct and indirect costs. The unit costs will be expressed in Euros (€) based on prices in 2013.

#### Utilities

These represent the rating of the patients’ quality of life on a scale from 0 (as bad as death) to 1 (perfect health). Negative values are possible indicating a health state that is “worse than death”. Patients describe their quality of life using the EQ-5D, which is preferred for economic evaluations from a societal perspective. Quality Adjusted Life Years (QALYs) are calculated using Spanish tariffs of EQ-5D.

#### Cost-utility analysis

Cost-utility is explored through the calculation of incremental cost-utility ratios (ICUR), defined as the difference in mean costs divided by difference in mean QALYs [55]. As the duration of the study is 12 months, neither costs nor outcomes are subject to discounting. QALYs gained in each evaluation are approximated by using the area under-the-curve technique. To gain insight into the uncertainty around the pooled mean ICUR, we will plot the pooled bootstrapped cost-effect pairs on cost-utility planes. Bootstrapping involves resampling with replacement from the original sample a sufficiently large number of times in order to approximate the distribution of the population from which the original data are drawn [56]. In our analyses, 1,000 samples will be generated. This distribution is used to calculate the probability that each of the treatments is the optimal choice, subject to a range of possible maximum values (ceiling ratio) that a decision-maker might be willing to pay for a unit improvement in outcome. Cost-utility acceptability curves are presented by plotting these probabilities for a range of possible values of the ceiling ratio [57]. The acceptability curve represents the probability that the intervention is cost-effective, given a varying threshold for the willingness to pay for each QALY gained. The curves obtained incorporate the uncertainty that exists around the estimates of expected costs and expected effects associated with the intervention [58].

We assume that data will be missing completely at random. Only patients with both cost and relevant outcome data at 12-month follow-up will be included in the cost-utility analyses. Notwithstanding, the robustness of the cost-utility results will be tested by also imputing missing 12-month data (sensitivity analysis). The imputations will be performed using the multiple imputation procedure in STATA 11.0.

### Forecast execution dates

Initial recruitment of patients: September 2012

Finalization of patient recruitment: December 2013

Finalization of patient monitoring period: June 2014

Publication of results: December 2014

## Discussion

The effectiveness of Internet-delivered psychotherapy for depression has been demonstrated [12,18]. However, the comparisons between low intensity and self-guided are infrequent, and also a comparative economic evaluation between them and compared with usual treatment in primary. The strength of the study is that, to our knowledge, this is the first multicenter, randomized, controlled trial of low intensity and self-guided Internet-delivered psychotherapy for depression in primary care, being the treatment completely integrated in primary care setting.

A number of potential limitations may be difficulties in recruitment, owing to negative attitudes of discontinue pharmacological treatment and changes in employment status because many patients are either on sick leave or applying for disability pensions, making it difficult to interpret the results.

### Clinical implications

This is probably the first study in Spain aiming to improve clinical status and quality of life of depressed patients in the community using an internet cognitive psychotherapy. If positive results are achieved, we cannot avoid the high impact this intervention would bring to the society. Moreover, if this therapy is efficient it means that it would be suitable for implementation from an economic point of view.

The treatment program proposed in this study includes therapeutic strategies that have proven their efficacy for depression, but it is also based on the rationale of transdiagnostic approaches that are emerging in the psychopathology and treatments fields. Such approaches are focused on fundamental processes underlying different disorders, helping to explain comorbidity among disorders, and leading to more effective assessment and treatment strategies. In the present study, the program targets the treatment of moderate depression and adjustment disorders. If the program efficacy is proven, then it could be used in other problems where depression or adjustment disorders are comorbid or common symptomatology is present. It may also have advantages in ease of dissemination and in treating other emotional disorders.

## Competing interests

The authors declare that they have no competing interests.

## Authors’ contributions

JGC, MR, MG, ASB, FC, FM, RB, CB, RM and RA are the principal researcher and developed the original idea for the study. The study design was further developed by BO, YLdH and JVL. All the authors participated in the design and planning of the intervention that is evaluated here. ASB developed the economic evaluation and JVL and EA the statistical methods. All authors have read and corrected draft versions, and approved the final version.

## Author’s information

Yolanda López-del-Hoyo, Barbara Olivan, Juan V. Luciano-Devis, Fermín Mayoral, Miquel Roca, Margalida Gili, Antoni Serrano-Blanco, Rosa Magallón, Javier García Campayo REDIAPP “Red de Investigación en Actividades Preventivas y Promoción de la Salud” (RD06/0018/0017).

## Pre-publication history

The pre-publication history for this paper can be accessed here:

http://www.biomedcentral.com/1471-244X/13/21/prepub

## References

[B1] Fernández A Pinto-Meza A Bellón JA Luciano JV Autonell J Palao D Salvador-Carulla L García Campayo J Haro JM Serrano-Blanco A DASMAP investigators Burden of chronic physical conditions and mental disorders in the Catalan Primary Care. Results from the DASMAP study Brit J Psychiat 2010 196 302 309 10.1192/bjp.bp.109.074211 20357307

[B2] Sartorius N Goldberg D Girolamo G Silva JA Ce Lecrubier Y Wittchen HU Psychological disorders in general medical settings 1990 New York: Hogrefe&Huber Publishers

[B3] World Health Organization The global burden of disease 2001 Géneve: WHO

[B4] Goldberg DP Huxley P Common mental disorders: A bio-social model 1992 London: Routledge

[B5] Serrano-Blanco A Palao DJ Luciano JV Pinto-Meza A Luján L Fernández A Roura P Bertsch J Mercader M Haro JM Prevalence of mental disorders in primary care: results from the Catalan ‘Diagnosis and Treatment of Mental Disorders in Primary Care Study Soc Psychiatry Psychiatr Epidemiol 2010 45 201 210 10.1007/s00127-009-0056-y 19452110

[B6] Van Schaik D Klijn A van Hout H Patients preferences in the treatment of depressive disorders in primary care Gen Hosp Psychiatry 2004 26 184 189 10.1016/j.genhosppsych.2003.12.001 15121346

[B7] Cuijpers P van Straten A van Oppen P Andersson G Are psychological and pharmacologic interventions equally effective in the treatment of adult depressive disorders? A meta-analysis of comparative studies J Clin Psychiatry 2008 69 1675 1685 10.4088/JCP.v69n1102 18945396

[B8] Andrews G Issakidis C Sanderson K Utilising survey data to inform public policy: comparison of the cost-effectiveness of treatment of ten mental disorders Br J Psychiatry 2004 184 526 533 10.1192/bjp.184.6.526 15172947

[B9] Lehtinen V Riikonen E Latineen E Promotion of Mental Health on the European Agenda. Report 2000 Finnish Ministry of Social Affairs and Health: Department for Prevention and Promotion

[B10] Kaltenthaler E Parry G Beverley C Ferriter M Computerized cognitive-behavioural therapy for depression: systematic review Br J Psychiatry 2008 193 181 184 10.1192/bjp.bp.106.025981 18757972

[B11] Marks IM Cavanagh K Gega L Computer-aided psychotherapy: revolution or bubble? Br J Psychiatry 2007 191 471 473 10.1192/bjp.bp.107.041152 18055948

[B12] Marks I Cavanagh K Computer-aided psychological treatments: evolving issues Annu Rev Clin Psychol 2009 5 121 141 10.1146/annurev.clinpsy.032408.153538 19327027

[B13] Andersson G Cuijpers P Internet-based and other computerized psychological treatments for adult depression: a meta-analysis Cogn Behav Ther 2009 38 196 205 10.1080/16506070903318960 20183695

[B14] Cuijpers P Mark IM van Straten A Computer-aided psychotherapy for anxiety disorders: a meta-analytic review Cogn Behav Ther 2009 38 66 82 10.1080/16506070802694776 20183688

[B15] Titov N Status of computerized cognitive behavioural therapy for adults Aust N Z J Psychiatry 2007 41 95 114 10.1080/00048670601109873 17464688

[B16] Bender JL Radhakrishnan A Diorio C Englesakis M Can pain be managed through the Internet? A systematic review of randomized controlled trials Pain 2011 152 1740 1750 10.1016/j.pain.2011.02.012 21565446

[B17] McCrone P Knapp M Proudfoot J Ryden C Cavanagh K Shapiro DA Ilson S Cost-effectiveness of computerized cognitive-behavioural therapy for anxiety and depression in primary care: randomised controlled trial Br J Psychiatry 2004 185 55 62 10.1192/bjp.185.1.55 15231556

[B18] McCrone P Marks IM Mataix-Cols D Kenwright M McDonough M Computer-Aided Self-Exposure Therapy for Phobia/Panic Disorder: A Pilot Economic Evaluation Cogn Behav Ther 2009 18 1 9 10.1080/16506070802561074 19306148

[B19] Hollinghurst S Peters TJ Kaur S Wiles N Lewis G Kessler D Cost-effectiveness of therapist-delivered online cognitive-behavioural therapy for depression: randomised controlled trial Br J Psychiatry 2010 197 297 304 10.1192/bjp.bp.109.073080 20884953

[B20] Gerhards SA de Graaf LE Jacobs LE Severens JL Huibers MJ Arntz A Economic evaluation of online computerized cognitive-behavioural therapy without support for depression in primary care: randomised trial Br J Psychiatry 2010 196 310 318 10.1192/bjp.bp.109.065748 20357309

[B21] National Institute for Health and Clinical Excellence Computerized cognitive behavioural therapy for depression and anxiety: Review of Technology Appraisal 51. Technology Appraisal 97 http://guidance.nice.org.uk/TA97

[B22] Learmonth D Rai S Taking computerized CBT beyond primary care Br J Clin Psychol 2008 47 111 118 10.1348/014466507X248599 17939879

[B23] Purves DG Bennett M Wellman N An open trial in the NHS of Blues Begone: a new home based computerized CBT program Behav Cogn Psychother 2009 37 541 551 10.1017/S1352465809990282 19703330

[B24] Cavanagh K Shapiro DA Van Den Berg S Swain S Barkham M Proudfoot J The effectiveness of computerized cognitive behavioural therapy in routine care Br J Clin Psychol 2006 45 499 514 10.1348/014466505X84782 17076960

[B25] Bennett-Levy J Richards DA Farrand P Bennett-Levy J, Richards DA, Farrand P Low intensity CBT interventions: a revolution in mental healthcare Oxford guide to low intensity CBT interventions 2010 Oxford: Oxford University Press 3 18

[B26] Haby MM Donnelly M Corry J Vos T Cognitive behavioural therapy for depression, panic disorder, and generalized anxiety disorder: a meta-regression of factors that may predict outcome Aust N Z J Psychiatry 2005 40 9 19 16403033 10.1080/j.1440-1614.2006.01736.x

[B27] Gellatly J Bower P Hennessy S What makes self-help interventions effective in the management of depressive symptoms? Meta-analysis and metaregression Psychol Med 2007 37 1217 1228 10.1017/S0033291707000062 17306044

[B28] Warmerdam L van Straten A Twisk J Internet-based treatment for adults with depressive symptoms: randomized controlled trial J Med Internet Res 2008 10 e44 10.2196/jmir.1094 19033149 PMC2629364

[B29] De Graaf LE Gerbards AH Arntz H Clinical effectiveness of online computerized cognitive–behavioural therapy without support for depression in primary care: randomised trial Br J Psychiatry 2009 195 73 80 10.1192/bjp.bp.108.054429 19567900

[B30] Waller R Gilbody S Barriers to the uptake of computerized cognitive behaviour therapy: a systematic review of the quantitative and qualitative evidency Psychol Med 2009 39 705 712 10.1017/S0033291708004224 18812006

[B31] Kaltenhthaler E Sutcliffe E Parry G Beverley C Rees A The acceptability to patients of computerized cognitive behaviour therapy for depression: a systematic review Psychol Med 2008 38 1521 1530 10.1017/S0033291707002607 18205964

[B32] Mitchell N Gordon PK Attitudes towards computerized CBT for depression amongst a student population Behav Cogn Psychother 2007 35 421 430 10.1017/S1352465807003700

[B33] Wangberg SC Gammon D Spitznogle K In the eyes of the beholder: exploring psychologists’ attitudes towards and use of e-therapy in Norway Cyberpsychol Behav 2007 10 418 422 10.1089/cpb.2006.9937 17594266

[B34] Beck AT Ester RA Ball R Ranieri W Comparison of Beck Depression Inventories -Ia and -II in psychiatric outpatients J Pers Assess 1996 67 588 597 10.1207/s15327752jpa6703_13 8991972

[B35] Sanz J Garcia Vera MP Espinosa R Fortun M Vazquez C Adaptación española del Inventario para la Depresión de Beck II: Propiedades psicométricas en pacientes con trastornos psicológicos Clínica y Salud 2005 16 121 14223573538

[B36] Bellón JA Moreno-Küstner B Torres-González F Montón-Franco C GildeGómez-Barragán MJ Sánchez-Celaya M Predicting the onset and persistence of episodes of depression in primary health care. The predictD-Spain study: methodology BMC Public Health 2008 8 256 10.1186/1471-2458-8-256 18657275 PMC2527330

[B37] Proudfoot J Ryden C Everitt B Shapiro DA Goldberg A Mann A Tylee A Marks I Gray JA Clinical efficacy of computerized cognitive-behavioural therapy for anxiety and depression in primary care: randomised controlled trial Br J Psychiatry 2004 185 46 54 10.1192/bjp.185.1.46 15231555

[B38] Roca M Gili M Garcia-Garcia M Salva J Vives M Garcia Campayo J Comas A Prevalence and comorbidity of common mental disorders in primary care J Affect Disord 2009 119 52 58 10.1016/j.jad.2009.03.014 19361865

[B39] García-Herrera JM Nogueras-Morillas V Muñoz-Cobos F Morales-Asensio JM Guía de Práctica Clínica para el Tratamiento de la Depresión en Atención Primaria [Clinical Practice Guide on the Treatment of Depression in Primary Care] 2011 Distrito Sanitario Málaga UGC Salud Mental Hospital Regional Universitario “Carlos Haya”: Málaga

[B40] Herrán A Rodriguez B Vazquez Barquero J Vazquez Barquero JL Psiquiatría en atención primaria 2007 Madrid: Aula Médica 222 250

[B41] Beecham J Knapp M Thornicroft G Costing psychiatric interventions Measuring Mental Health Needs 2001 London: Gaskell

[B42] Lecrubier Y Sheehan D Weiller E Amorim P Bonora I Sheehan K Janavs J Dunbar G The Mini International Neuropsychiatric Interview (M.I.N.I.), a short diagnostic interview: Reliability and validity according to the CIDI Eur Psychiatry 1997 12 232 241 10.1016/S0924-9338(97)83297-X

[B43] Ferrando L Franco L Soto M Bobes J Soto O Franco L Gibert J MINI. MINI International Neuropsychiatric Interview. Versión en español 5.0.0 1998 Madrid: IAP

[B44] Badia X EuroQol; un instrumento para valorar la salud EQ-5D guía del usuario, version española Med Clin (Barc) 1999 114 6 14

[B45] Badia X Roset M Montserrat S Herdman M Segura A The Spanish version of EuroQol: a description and its applications. European Quality of Life scale Med Clin (Barc) 1999 112 Suppl 1 79 85 10618804

[B46] Knapp M PSSRU and Centre for Economics of Mental Health, Institute of Psychiatry, University of Kent Economic Evaluation of Mental Health Care 1995 Kent, GB: Ashgate Publishing Group

[B47] Norman SB Cissell SH Means-Christensen AJ Stein MB Development and validation of an Overall Anxiety Severity And Impairment Scale (OASIS) Depres Anxiety 2006 23 245 249 10.1002/da.20182 16688739

[B48] Watson D Clark L Tellegen A Development and validation of brief measures of positive and negative affect: The PANAS scales J Personality Social Psychol 1988 54 1063 1070 10.1037//0022-3514.54.6.1063 3397865

[B49] Sandin B Chorot P Lostao L Joiner T Santed M Valiente R Escalas PANAS de afecto positivo y negativo: Validacion factorial y convergencia transcultural Psicothema 1999 11 37 51

[B50] Borkovec TD Nau SD Credibility of analogue therapy rationales J Behav Ther Exp Psychiatry 1972 3 257 260 10.1016/0005-7916(72)90045-6

[B51] Kazdin AE Therapy outcome questions requiring control of credibility and treatment-generated expectancies Behav Therapy 1979 10 81 93 10.1016/S0005-7894(79)80011-8

[B52] Devilly GJ Borkovec TD Psychometric properties of the credibility/expectancy questionnaire J Behav Ther Exp Psychiatry 2000 31 2 73 86 10.1016/S0005-7916(00)00012-4 11132119

[B53] Baños RM Botella C Guillén V García-Palacios A Jorquera M Quero S Un programa de tratamiento para los trastornos adaptativos: Un estudio de caso Apunt Psicología 2008 26 303 31615006166

[B54] Schulz KF Altman DG Moher D CONSORT Group CONSORT 2010 Statement: updated guidelines for reporting parallel group randomised trials Ann Int Med 2010 152 726 732 20335313 10.7326/0003-4819-152-11-201006010-00232

[B55] Van Hout BA Al MJ Gordon GS Costs, effects and C/E-ratios alongside a clinical trial Health Econ 1994 3 309 319 10.1002/hec.4730030505 7827647

[B56] Mooney C Duval R Bootstrapping: A Non-parametric Approach to Statistical Inference 1993 London: Sage

[B57] Fenwick E Claxton K Sculpher M Representing uncertainty: The role of cost-effectiveness acceptability curves Health Econ 2001 10 779 787 10.1002/hec.635 11747057

[B58] Fenwick E Byford S A guide to cost-effectiveness acceptability curves Br J Psychiatry 2005 187 106 108 10.1192/bjp.187.2.106 16055820

